# LIVED EXPERIENCES OF OLDER FEMALE SEX WORKERS AT SONAGACHI, KOLKATA: A CASE STUDY

**DOI:** 10.1093/geroni/igad104.2328

**Published:** 2023-12-21

**Authors:** Esha Chakravarty, Indrani Chakravarty

**Affiliations:** Calcutta Metropolitan Institute of Gerontology, Mitcham, England, United Kingdom; Calcutta Metropolitan Institute of Gerontology, Kolkata, West Bengal, India

## Abstract

Older sex workers are marginalised because of their former professional engagement and old age. This study captured the experiences of older female sex workers (FSWs), aged sixty and above, who delivered sexual services at Sonagachi, Asia’s largest pleasure market. Exploratory research design was used to understand their lived experiences through a qualitative intervention. The study was content driven, not hypothesis driven. Six older FSWs were selected through purposive sampling, primary data was collected through case study method. Their experiences were recorded starting from childhood, entry into sex trade, daily professional challenges and experiences during old age. Data collected was thematically analysed. Older Sonagachi FSWs are a heterogeneous group in terms of socio-demographic variables, health condition, place and nature of work, average load of clients they entertained per day. This study found that economic adversities and poverty during growing years have been the foremost reasons for choosing sex trade. Many of them remained in the sex industry because of lack of alternative professional opportunities. They experienced various forms of community perpetrated exploitation and some had acquired sexually transmitted infections. Other reasons for them to be in this trade were childhood poverty, early marriage, early motherhood, lack of education, lack of employment, neglect and abuse. They were destitute, had no caregivers, struggled to be part of mainstream society. The study suggests the need to develop policies for vocationally training them to generate alternative income sources and to initiate welfare schemes including pension plans and developing separate old age homes for older FSWs.

